# Effect of Different Piperacillin-Tazobactam Dosage Regimens on Synergy of the Combination with Tobramycin against *Pseudomonas aeruginosa* for the Pharmacokinetics of Critically Ill Patients in a Dynamic Infection Model

**DOI:** 10.3390/antibiotics11010101

**Published:** 2022-01-13

**Authors:** Jessica R. Tait, Hajira Bilal, Kate E. Rogers, Yinzhi Lang, Tae-Hwan Kim, Jieqiang Zhou, Steven C. Wallis, Jürgen B. Bulitta, Carl M. J. Kirkpatrick, David L. Paterson, Jeffrey Lipman, Phillip J. Bergen, Jason A. Roberts, Roger L. Nation, Cornelia B. Landersdorfer

**Affiliations:** 1 Drug Delivery, Disposition and Dynamics, Monash Institute of Pharmaceutical Sciences, Monash University, Parkville, VIC 3052, Australia; Jessica.Tait@monash.edu (J.R.T.); Kate.Rogers@monash.edu (K.E.R.); Roger.Nation@monash.edu (R.L.N.); 2 Centre for Medicine Use and Safety, Monash Institute of Pharmaceutical Sciences, Monash University, Parkville, VIC 3052, Australia; hajira.bilal1@monash.edu (H.B.); carl.kirkpatrick@monash.edu (C.M.J.K.); phillip.bergen@monash.edu (P.J.B.); 3 Department of Pharmacotherapy and Translational Research, College of Pharmacy, University of Florida, Orlando, FL 32827, USA; y.lang@cop.ufl.edu (Y.L.); zhou.jieqiang@cop.ufl.edu (J.Z.); JBulitta@cop.ufl.edu (J.B.B.); 4 College of Pharmacy, Daegu Catholic University, Gyeongsan 38430, Korea; taehwan.sch@gmail.com; 5 The University of Queensland Center for Clinical Research, The University of Queensland, Brisbane, QLD 4029, Australia; s.wallis@uq.edu.au (S.C.W.); david.antibiotics@gmail.com (D.L.P.); j.lipman@uq.edu.au (J.L.); j.roberts2@uq.edu.au (J.A.R.); 6 Intensive Care Unit, Royal Brisbane and Women’s Hospital, Brisbane, QLD 4029, Australia; 7 Division of Anaesthesiology Critical Care Emergency and Pain Medicine, Nîmes University Hospital, University of Montpellier, 30900 Nîmes, France; 8 Jamieson Trauma Institute, Royal Brisbane and Women’s Hospital, Brisbane, QLD 4029, Australia

**Keywords:** dynamic infection model, *Pseudomonas aeruginosa*, pharmacokinetics, pharmacodynamics

## Abstract

We evaluated piperacillin-tazobactam and tobramycin regimens against *Pseudomonas aeruginosa* isolates from critically ill patients. Static-concentration time-kill studies (SCTK) assessed piperacillin-tazobactam and tobramycin monotherapies and combinations against four isolates over 72 h. A 120 h-dynamic in vitro infection model (IVM) investigated isolates Pa1281 (MIC_piperacillin_ 4 mg/L, MIC_tobramycin_ 0.5 mg/L) and CR380 (MIC_piperacillin_ 32 mg/L, MIC_tobramycin_ 1 mg/L), simulating the pharmacokinetics of: (A) tobramycin 7 mg/kg q24 h (0.5 h-infusions, t_1/2_ = 3.1 h); (B) piperacillin 4 g q4 h (0.5 h-infusions, t_1/2_ = 1.5 h); (C) piperacillin 24 g/day, continuous infusion; A + B; A + C. Total and less-susceptible bacteria were determined. SCTK demonstrated synergy of the combination for all isolates. In the IVM, regimens A and B provided initial killing, followed by extensive regrowth by 72 h for both isolates. C provided >4 log_10_ CFU/mL killing, followed by regrowth close to initial inoculum by 96 h for Pa1281, and suppressed growth to <4 log_10_ CFU/mL for CR380. A and A + B initially suppressed counts of both isolates to <1 log_10_ CFU/mL, before regrowth to control or starting inoculum and resistance emergence by 72 h. Overall, the combination including intermittent piperacillin-tazobactam did not provide a benefit over tobramycin monotherapy. A + C, the combination regimen with continuous infusion of piperacillin-tazobactam, provided synergistic killing (counts <1 log_10_ CFU/mL) of Pa1281 and CR380, and suppressed regrowth to <2 and <4 log_10_ CFU/mL, respectively, and resistance emergence over 120 h. The shape of the concentration–time curve was important for synergy of the combination.

## 1. Introduction

Serious infections caused by *Pseudomonas aeruginosa*, such as bacteremia and pneumonia, are posing a significant challenge to patients in intensive care units (ICUs) and are associated with high rates of morbidity and mortality [1,2,3]. In Europe, *P. aeruginosa* has been reported to be the most frequently isolated microorganism in episodes of ICU-acquired pneumonia and the second most common Gram-negative pathogen isolated in ICU-acquired bacteremia [4]. In the United States, multidrug-resistant (MDR) *P. aeruginosa* strains cause an estimated 32,600 infections per year in hospitalized patients [5]. Patients with *P. aeruginosa* infections have a higher mortality rate than those infected by other Gram-negative pathogens [6]. *P. aeruginosa* is intrinsically resistant to many antibiotics and has a particularly high propensity to develop resistance to all available antipseudomonals [7,8]. Suboptimal antibiotic exposures increase the risk of resistance emergence and therapeutic failure [9,10]. Critically ill patients are particularly vulnerable to such treatment failures due to physiological and pharmacokinetic changes, which may require the clinical use of higher than standard dosing regimens, including off-label dosages [11,12].

Early initiation of effective antimicrobial therapy is associated with a substantially improved probability of survival in sepsis and septic shock, as demonstrated in multiple studies and meta-analyses [13,14,15]. Consequently, it has been recommended that antipseudomonal therapy of serious infections should be initiated with two agents from different classes, such as a β-lactam and an aminoglycoside, especially in settings with a high risk of resistance [16]. In addition to the choice of antibiotics, the importance of optimized dosage regimens to achieve adequate exposure-response profiles has been emphasized [16,17,18]. These recommendations indicate that it is important to initiate antibiotic therapy as early as possible with an optimized combination regimen.

Piperacillin-tazobactam and tobramycin are commonly used antibiotics against serious *P. aeruginosa* infections. Based on pharmacokinetic/pharmacodynamic (PK/PD) principles, the shape of the concentration-time profile has the potential to impact antibacterial effects for a range of antibiotics, including β-lactams [19,20]. Population PK modeling and simulations indicated that, compared to standard 8-hourly short-term infusions, continuous infusion of the same daily dose of piperacillin-tazobactam increased the probability of attaining therapeutic plasma concentrations in the early phase of septic shock [21]. Based on multiple meta-analyses, both continuous infusion and intermittent prolonged infusions of β-lactams, including piperacillin-tazobactam, were associated with decreased hospital mortality and/or increased clinical cure [22,23,24,25,26]. We have previously shown that piperacillin-tazobactam administered via intermittent infusions in combination with tobramycin was synergistic against a piperacillin- and tobramycin-susceptible *P. aeruginosa* ICU isolate for the PK of critically ill patients with augmented renal clearance [27]. However, that study did not compare different modes of administration and thus shapes of the piperacillin concentration-time profile. Additionally, the effect of different modes of administration of piperacillin (such as continuous compared to short-term infusions) in combination with tobramycin has not been evaluated against isolates representing a range of susceptibilities to piperacillin and for the PK of critically ill patients with normal renal function.

Our first objective was to quantify, in static-concentration time-kill experiments (SCTK), bacterial killing and suppression of less-susceptible subpopulations for combinations of piperacillin-tazobactam and tobramycin against four *P. aeruginosa* ICU isolates with a range of different susceptibilities to piperacillin-tazobactam and including multidrug-resistant (MDR) isolates. The second objective was to evaluate the effect of different dosage regimens of piperacillin-tazobactam, alone and in combination with tobramycin, on bacterial killing and emergence of less-susceptible subpopulations of two of the isolates in a dynamic in vitro infection model (IVM).

## 2. Results

Key characteristics of the four isolates are summarized in Table 1. The isolates had tobramycin MICs of 0.5 mg/L or 1 mg/L and piperacillin-tazobactam MICs ranging from 4 mg/L to 32 mg/L. Three isolates were carbapenem-resistant, with two of those MDR. For the piperacillin-tazobactam static and dynamic studies below, the concentrations and doses refer to the piperacillin component.

### 2.1. Static-Concentration Time-Kill Studies

Log changes from SCTK studies are presented in Table S1, where green and blue highlighting indicate synergistic and enhanced bacterial killing, respectively. At the low inoculum (~10^6^ CFU/mL), tobramycin monotherapies of 2 mg/L and 8 mg/L resulted in substantial reductions in bacterial numbers against all four isolates. For the piperacillin-tazobactam monotherapies against the low inoculum, it was only the highest piperacillin concentration (75 mg/L) that resulted in a reduction in bacterial density either all or the majority of times for each isolate; the magnitude of the reduction was inversely associated with the MIC. Regrowth after initial bacterial killing was a feature of monotherapy with either piperacillin or tobramycin against each of the isolates at the low inoculum (Table S1). At the high inoculum (~10^7.5^ CFU/mL), the antibacterial effects of the respective treatments were generally smaller than at the low inoculum. Combinations provided effective and often synergistic or enhanced killing of each isolate at both inocula (Table S1), with greater suppression of regrowth at the higher antibiotic concentrations. An inoculum effect was also observed in combinations against isolates CR379, CR380, and CR382, whereby growth by 72 h was approximately 2 log_10_ higher in the high compared to the low inoculum for almost all combinations with tobramycin concentrations less than 8 mg/L. Against Pa1281, which had the lowest MICs against both antibiotics among the set of isolates tested, all combinations suppressed growth below the limit of counting, except for piperacillin 12 mg/L plus tobramycin 1 mg/L at the high inoculum.

### 2.2. Dynamic In Vitro Infection Model

**Figure 1:**
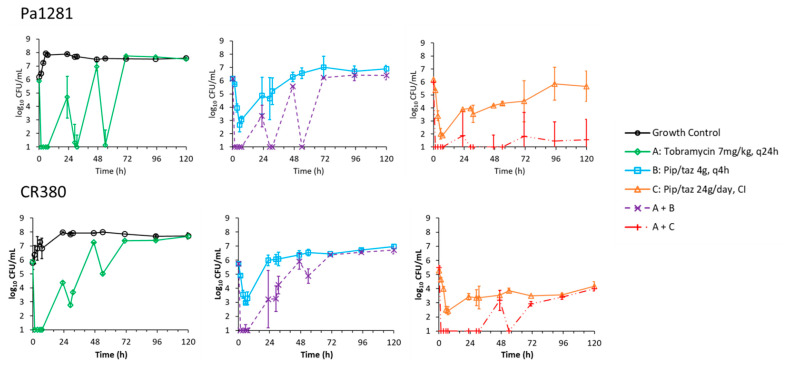
Counts of total viable bacteria (average ± SE ^a^) from the dynamic in vitro infection model of *P. aeruginosa* clinical isolates Pa1281 and CR380 against piperacillin-tazobactam (pip/taz) and tobramycin, alone and in combination. Observations below the limit of counting (1.0 log_10_ CFU/mL) are plotted at 1.0 log_10_ CFU/mL. ^a^ Performed in biological replicates, *n* = 2 except for CR380 vs. B and A + B where *n* = 3.

**Figure 2:**
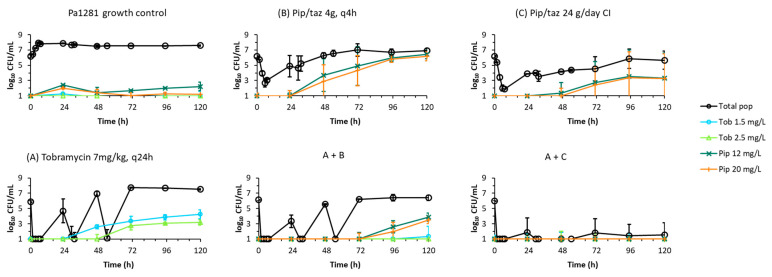
Less susceptible subpopulations of clinical isolate Pa1281 (average ± SE, *n* = 2) in the dynamic in vitro infection model quantified on antibiotic-containing agar plates. The total populations (from antibiotic-free agar plates) in each panel below are as shown in Figure 1. Observations below the limit of counting (0.7 log_10_ CFU/mL) are plotted at 1.0 log_10_ CFU/mL.

**Figure 3:**
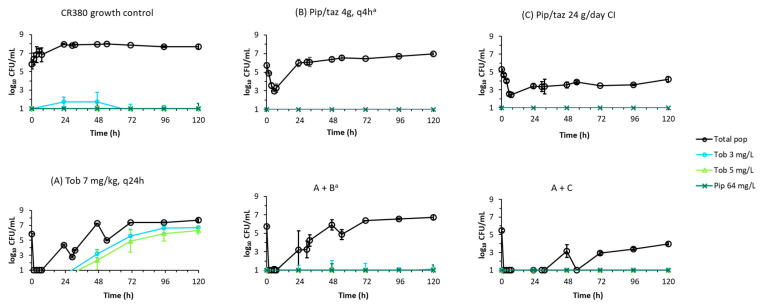
Less susceptible subpopulations of CR380 (average ± SE ^a^) in the dynamic in vitro infection model detected on antibiotic-containing agar plates. The total population is representative of what is shown in Figure 1. Observations below the limit of counting (0.7 log_10_ CFU/mL) are plotted at 1.0 log_10_ CFU/mL. ^a^ Performed in biological replicates, *n* = 2, except for treatment arms B and A + B where *n* = 3.

Measured concentrations of piperacillin and tobramycin in the dynamic IVM were on average within 5.4% of targeted concentrations (plotted in Figure S1). Counts of viable bacteria from the dynamic IVM for isolates Pa1281 and CR380 are plotted in Figure 1 and counts from antibiotic-containing agar plates are plotted in Figure 2 and Figure 3 for Pa1281 and CR380, respectively. Log changes are presented in Table 2.

All monotherapies failed in the dynamic IVM, where neither of the antibiotics provided complete suppression of regrowth. Tobramycin monotherapy (7 mg/kg once daily) failed against both isolates, with regrowth of the total bacterial population to control levels by 72 h and less-susceptible subpopulations evident from 48–72 h onwards (Figure 1, Figure 2 and Figure 3). A greater extent of killing after dosing at 24 and 48 h was observed against Pa1281 (up to ~5 log_10_ killing) compared to CR380 (up to ~2.2 log_10_ killing). Against Pa1281, both the intermittent (4 g every 4 h) and continuous infusion (24 g per day) dosing regimens of piperacillin-tazobactam provided initial killing of up to ~4 log_10_ CFU/mL. This was followed by regrowth of the total population to 6.9 and 5.7 log_10_ CFU/mL by 120 h for the intermittent and continuous infusion, respectively, corresponding to bacterial densities approximately 1 and 2 log_10_ below the growth control. Less-susceptible subpopulations were observed from 48 h. A more pronounced difference in regrowth between the two piperacillin-tazobactam regimens was observed for CR380, where the continuous infusion was able to suppress growth of the total population to below ~4 log_10_ CFU/mL throughout, while regrowth to ~7 log_10_ CFU/mL by 120 h occurred with the intermittent infusion regimen. Subpopulations less susceptible to piperacillin were not observed for isolate CR380.

For each isolate, the shape of the time–course of the bacterial counts of the total population for the combination of intermittent piperacillin and tobramycin was very similar to that of tobramycin monotherapy over the first 54 h, but the bacterial densities at each time point were up to 1.5 log_10_ lower for the combination regimen (Figure 1). Enhanced killing was only observed at a relatively small number of times, but there were no instances of synergistic killing (Table 2). By 120 h, regrowth to bacterial counts approximately 0.25 to 0.5 log_10_ lower than those of the corresponding piperacillin monotherapy profiles was observed for both isolates. For Pa1281, subpopulations less susceptible to piperacillin were amplified by the intermittent combination regimen, compared to control levels, from 96 h onwards (Figure 2). Low levels of subpopulations less susceptible to tobramycin were detected in CR380 from 96 h (Figure 3).

Piperacillin-tazobactam delivered via continuous infusion in combination with tobramycin administered intermittently provided synergistic killing and more extensive suppression of regrowth of the total population and of less-susceptible subpopulations against both isolates, compared to the intermittent combination regimen (Figure 1, Figure 2 and Figure 3, Table 2). For Pa1281, growth of the total population was suppressed to below ~2 log_10_ CFU/mL from 1.5 to 120 h. In the case of isolate CR380, the greatest suppression of regrowth occurred up to 47 h; from 72 h onward, the combination regimen resulted in slightly lower total viable counts than for its corresponding piperacillin monotherapy. Subpopulations less susceptible to either antibiotic were not detected for either isolate for this combination.

## 3. Discussion

In the studies described herein, we explored the activity of combinations of piperacillin-tazobactam and tobramycin at clinically relevant concentrations [29,30] against clinical ICU isolates of *P. aeruginosa* in static and dynamic in vitro models. The concentration versus time exposure profiles simulated in the dynamic model were based on the PK of critically ill patients with normal renal function receiving clinically relevant daily doses of piperacillin-tazobactam and tobramycin [29,31,32,33,34,35]. In the SCTK studies, synergy or enhanced activity was observed at both low and high inocula against all four isolates which had piperacillin-tazobactam and tobramycin MICs of 4–32 mg/L and 0.5–1 mg/L, respectively. These results are in keeping with previous studies that have reported synergistic activity of piperacillin and tobramycin combinations against *P. aeruginosa* in static in vitro systems [27,36,37,38,39,40]. In the studies conducted in the dynamic in vitro infection model, against two isolates with the lowest and highest MICs of piperacillin and tobramycin, we observed that intermittent administration of piperacillin-tazobactam every 4 h in combination with once-daily administration of tobramycin was not synergistic and resulted in regrowth with resistance by 120 h. The combination of tobramycin administered once daily plus piperacillin-tazobactam via continuous infusion was required for a substantial reduction in bacterial load and synergistic killing. Resistance emergence was not observed for either isolate for this combination regimen.

The divergent responses achieved in the dynamic in vitro model between the combinations involving the two different modes of administration of piperacillin-tazobactam are consistent with previous studies that have demonstrated the impact of the shape of the exposure profile of an antibiotic on the resultant antibacterial response [41,42,43,44,45,46]. The marked reduction in bacterial load, synergy, and suppression of resistance achieved in the dynamic model for the combination of tobramycin administered once daily plus piperacillin-tazobactam via continuous infusion are consistent with required qualities of appropriate initial antibiotic treatment [47].

For β-lactams, the PK/PD target considered necessary to maximize the likelihood of successful treatment has evolved in recent years. Initially it was thought that attaining a substantial proportion of time, e.g., 40–70%, where the free (i.e., unbound) concentration was above the MIC (*f*T_>MIC_) was an appropriate target [48]. Over time, the application of a target of 100% *f*T_>MIC_ has become more prevalent for achievement of optimal bacterial killing and resistance suppression for difficult-to-treat pathogens and critically ill patients [49,50]. Recently, some studies have explored even higher targets, e.g., 100% *f*T_>4× MIC_ [51,52]. In the current dynamic in vitro study, the continuous infusion of piperacillin-tazobactam alone provided an exposure of 100% *f*T_>10× MIC_ for the isolate with the lowest piperacillin-tazobactam MIC (Pa1281) (Table 3). However, even with such a high daily dose and thus high level of exposure, that monotherapy regimen still resulted in a mid to high bacterial density with resistance amplification at the conclusion of the study at 120 h, confirmed in biological replicates. Similarly, tobramycin monotherapy failed against both isolates in the dynamic model with extensive bacterial regrowth and amplification of less-susceptible populations, even though the traditional tobramycin PK/PD targets (i.e., ratio of free maximal concentration to MIC (*f*C_max_/MIC) of 8–10 and ratio of free area under the curve to MIC (*f*AUC/MIC) >70) [11] were reached (Table 3). Despite the 2-fold and 8-fold differences in the MICs of tobramycin and piperacillin-tazobactam, respectively, between isolate Pa1281 and CR380, there was little difference in the respective time-courses of the total bacterial population across 120 h with any of the monotherapy regimens. This reinforces the increasing awareness and concern around the limitations of MIC measurements as a guide to antimicrobial chemotherapy and of the applicability of the traditional PK/PD indices (*f*T_>MIC_, *f*C_max_/MIC and *f*AUC/MIC) and the associated exposure targets [17,45,53,54,55,56,57].

Synergy between piperacillin-tazobactam and tobramycin was observed for all four isolates in the SCTK and for the combination regimen including piperacillin-tazobactam as continuous infusion with both isolates in the dynamic IVM. This observation might be attributed to the different mechanisms of action and of resistance of β-lactams and aminoglycosides [27]. Piperacillin inhibits cell wall synthesis via binding to penicillin-binding proteins (PBPs). Mechanisms of resistance to piperacillin-tazobactam in *P. aeruginosa* include overexpression of chromosomally mediated AmpC β-lactamase and the MexAB-OprM efflux system [58,59]. Tobramycin blocks protein synthesis, but also disrupts the outer bacterial membrane [60,61]. Resistance mechanisms of *P. aeruginosa* against aminoglycosides include increased expression of the MexXY-OprM efflux system, target-site modification, enzymatic cleavage, and reduced outer membrane permeability. Another likely reason for the observed synergy is that disruption of the bacterial outer membrane by tobramycin may result in increased piperacillin concentrations in the periplasmic space where the PBPs are located [60,62]. However, tobramycin and piperacillin also share a resistance mechanism, whereby sub-inhibitory concentrations of tobramycin can induce the MexXY-OprM efflux system, which also affects piperacillin [63,64,65,66]. This may have contributed to the regrowth in response to the combination including intermittent piperacillin. This could not be confirmed, as genomic analysis of recovered colonies was outside the scope of this study.

This study has a number of strengths. The SCTK included a range of bacterial strains with differing susceptibility profiles and two starting inocula. Additionally, the results from the dynamic IVM studies were confirmed in biological replicates for two isolates, and both the total bacterial population and the less-susceptible subpopulations were quantified. Additionally, it is the first in vitro experiment to study piperacillin-tazobactam as a continuous infusion in combination with intermittent tobramycin against *P. aeruginosa.* A limitation of this work is that the static and dynamic in vitro infection models used in this study exclude the role of an immune response, thus demonstrating likely scenarios in immunocompromised patients. Further investigation with an in vivo model is warranted to provide insight on the role of the immune system in eradicating infections once bacterial density drops to low levels, as was seen here induced by tobramycin administered once daily in combination with piperacillin-tazobactam as a continuous infusion against both isolates with differing susceptibilities.

In conclusion, the static and dynamic studies described here demonstrated that the combination of piperacillin-tazobactam and tobramycin resulted in synergistic killing of *P. aeruginosa* isolates, including MDR and carbapenem-resistant strains, with a range of susceptibilities to piperacillin-tazobactam. The studies with the combination in the dynamic in vitro infection model involving the administration of the same daily dose of piperacillin-tazobactam administered in two different ways (intermittent versus continuous infusions) highlighted the importance of optimizing not only the dose of the β-lactam, but also the way in which it is administered. The combination with piperacillin-tazobactam delivered via continuous infusion resulted in synergistic killing and more extensive suppression of regrowth of the total population and of less-susceptible subpopulations, compared to the intermittent combination regimen.

## 4. Materials and Methods

### 4.1. Bacterial Isolates, Antibiotics, Media and Susceptibility Testing

*P. aeruginosa* clinical isolates CR379, CR380, CR382, and Pa1281 were from critically ill patients (Royal Brisbane and Women’s Hospital, Brisbane, QLD, Australia). Piperacillin-tazobactam was purchased from Sandoz Pty Ltd., NSW, Australia (4 g of piperacillin-0.5 g of tazobactam per vial); throughout this report, the stated doses and concentrations refer to those of piperacillin. Tobramycin from AK Scientific Inc., Union City, CA, was used for all studies. Stock solutions were prepared in distilled water and filter sterilized by use of a Millex-GV 0.22-µm polyvinylidene difluoride syringe filter (Merck Millipore Ltd., Cork, Ireland). Stocks were stored at −80 °C and thawed immediately prior to each experiment. Cation-adjusted Mueller Hinton broth (CAMHB) and cation-adjusted Mueller Hinton agar (CAMHA) (Becton Dickinson & Co., Sparks, MD, USA, with 25.0 mg/L Ca^2+^ and 12.5 mg/L Mg^2+^) were used in all studies. Minimum inhibitory concentrations (MIC) were determined for each isolate in triplicate via agar dilution (Table 1) [67].

### 4.2. Static-Concentration Time-Kill Experiments

For each isolate at low and high inocula (~10^6^ and ~10^7.5^ CFU/mL, respectively), piperacillin-tazobactam and tobramycin were studied as monotherapies and in combination in SCTK studies over 72 h (32 treatment and control arms per isolate), performed as previously described [27]. Concentrations of each antibiotic were chosen to be in the range of clinically achievable unbound plasma concentrations for critically ill patients following typical daily doses [29,30]. At 24 and 48 h, bacterial suspensions were centrifuged (10 min at 3220× *g* and 36 °C), the supernatant carefully removed, and bacteria resuspended in sterile, prewarmed CAMHB containing the targeted antibiotic concentrations, to compensate for the thermal degradation of piperacillin-tazobactam [27,68]. Total viable count samples were collected at 0 h (pre-dose), and at 1.5, 3, 6, 24, 29, 48, and 72 h. Bacterial samples were washed twice in sterile saline. Serial dilution was performed by the addition of 100 µL of undiluted bacterial suspension to 900 µL of sterile saline. Viable counts were determined by manually plating 100 µL of an undiluted or appropriately diluted suspension in saline onto CAMHA plates [69]. Agar plates were incubated at 36 °C for 24 h, and colonies counted manually.

### 4.3. Dynamic In Vitro Infection Model

A one-compartment dynamic IVM was used to investigate two dosing regimens of piperacillin-tazobactam, each in combination with tobramycin. Tobramycin was administered to simulate predicted plasma concentrations arising from administration of 7 mg/kg every 24 h as 30 min infusions (A) [31,34]. Piperacillin-tazobactam was administered to simulate the predicted piperacillin plasma concentrations of an intermittent regimen of 4 g, as 30 min infusions dosed 4-hourly (B), and the equivalent daily dose of 24 g/day via continuous infusion (C) [31,32,33,70,71,72]. Each piperacillin regimen was studied alone and in combination with tobramycin (i.e., A, B, C, A + B, and A + C), along with a growth control, in biological replicates. Targeted pharmacokinetic profiles of concentrations in the IVM (Table 3) were generated with Berkeley Madonna (v8.3.18) based on published population PK models of critically ill patients, with normal renal clearance [29,35].

Two isolates were selected for testing in the IVM. The first was Pa1281 (piperacillin-tazobactam MIC 4 mg/L) and the second CR380 (piperacillin-tazobactam MIC 32 mg/L; carbapenem-resistant and MDR). The tobramycin MIC was 0.5 mg/L for Pa1281 and 1 mg/L for CR380. Each isolate was examined in the IVM over 120 h, as previously described [73]. Briefly, a prepared bacterial suspension was injected into the media within the central reservoir immediately prior to antibiotic treatment to achieve an initial inoculum of ~10^6^ CFU/mL. For a regimen with piperacillin-tazobactam as continuous infusion, the central reservoir was prepared with media dosed to the target piperacillin concentration prior to initiation of the experiment. The concentration was maintained by adding the appropriate dose of piperacillin-tazobactam to the diluent medium, which was replaced daily. The intermittent regimens of piperacillin (t_1/2_ = 1.5 h) and tobramycin (t_1/2_ = 3.1 h) were infused over 30 min via separate syringe drivers at the dosing intervals described above. For regimens with intermittent piperacillin, a bolus dose was employed to achieve the steady-state trough concentration at 0 h (Table 3). The flow rate of media through the system was set to achieve the half-life of piperacillin (1.5 h). Tobramycin was supplemented appropriately over time by an additional syringe driver to enable simulating the differing half-lives of each antibiotic studied [44,74].

Samples were collected from the central reservoir at 0, 1.5, 3.5, 5.5, 7, 23, 29, 31, 47, 54, 71, 95, and 120 h for counting of viable bacteria. Samples were washed twice with saline to minimize antibiotic carry-over, serially diluted in saline, and plated on CAMHA for viable counting. Samples at 0, 23, 47, 71, 95, and 120 h were also plated on antibiotic-containing CAMHA to determine less-susceptible subpopulations. Antibiotic-free CAMHA plates were incubated at 36 °C for 24 h, and antibiotic-containing CAMHA plates (concentrations selected from 2×, 3×, and 5× MIC of the respective isolate) for 48 h.

Pharmacokinetic samples were collected at 0.66, 1.5, 3.5, 5.5, 7, 8.66, 23, 24.66, 31, 48.66, 54, 72.66, and 96.66 h. Validated LC-MS/MS assays were used to analyze concentrations of piperacillin and tobramycin in pharmacokinetic samples, in batches alongside matrix-matched calibrators and quality control samples [73,74,75]. Assay performance met batch acceptance criteria [76]. Precision and accuracy were within 7.6% and 4.7% for piperacillin, and within 5.8% and 14.3% for tobramycin, respectively.

### 4.4. Pharmacodynamic Analysis

The log-change method to assess the pharmacodynamic response to treatments was used for SCTK and IVM studies. Log changes in total viable bacteria were calculated to compare the change in log_10_ CFU/mL from 0 h (CFU_0_) to time t (CFU_t_), where log change = log_10_ (CFU_t_) − log_10_ (CFU_0_). Synergy with a combination regimen was defined as ≥2 log_10_ bacterial killing for the combination relative to its most active component at the specified time and ≥2 log_10_ below the initial inoculum. Enhanced activity was defined as a 1 to <2 log_10_ superior killing for the combination compared to its most active component at the specified time and ≥2 log_10_ below the initial inoculum.

## Figures and Tables

**Table 1:** Minimum inhibitory concentrations (MIC, mg/L) of *P. aeruginosa* isolates for piperacillin-tazobactam and tobramycin.

Isolate	Piperacillin-Tazobactam MIC	Tobramycin MIC	Comment
Pa1281	4	0.5	
CR382	16	1	CR
CR379	32	1	CR, MDR
CR380	32	1	CR, MDR

MDR: multidrug-resistant (non-susceptible to at least one agent in three or more antimicrobial categories [28]) based on CLSI breakpoints, CR: carbapenem-resistant.

**Table 2:** Log change for each treatment as a function of time from the dynamic in vitro infection model.

Isolate	Time (h)	A: Tob 7 mg/kg, q24 h	B: Pip/taz 4 g, q4 h	C: Pip/taz 24 g/day, CI	A + B	A + C
CR380	1.5	−5.86	−0.86	−0.62	−5.75	−5.50
	3.5	−5.86	−2.18	−1.29	−5.75	−5.50
	5.5	−5.86	−2.78	−2.75	−5.42	−5.50
	7	−5.86	−2.47	−2.84	−5.75	−5.50
	23	−1.50	0.24	−1.85	−2.53	−5.50
	29	−3.09	0.31	−1.92	−2.50	−5.50
	31	−2.18	0.34	−1.91	−1.52	−5.50
	47	1.40	0.64	−1.73	0.16	−2.33
	54	−0.84	0.79	−1.42	−0.88	−5.50
	71	1.51	0.70	−1.79	0.63	−2.57
	95	1.53	0.97	−1.71	0.81	−2.10
	120	1.82	1.22	−1.11	0.97	−1.51
Pa1281	1.5	−5.89	−0.44	−0.81	−6.13	−5.97
	3.5	−5.89	−2.24	−2.79	−6.13	−5.97
	5.5	−5.89	−3.51	−4.18	−6.13	−5.97
	7	−5.89	−3.12	−4.29	−6.13	−5.97
	23	−1.20	−1.29	−2.30	−2.79	−4.10
	29	−4.56	−1.53	−2.21	−6.13	−5.97
	31	−5.00	−0.96	−2.65	−6.13	−5.97
	47	1.07	0.11	−2.01	−0.55	−5.05
	54	−4.76	0.40	−1.84	−6.13	−5.97
	71	1.85	0.84	−1.67	0.10	−4.15
	95	1.79	0.54	−0.33	0.30	−4.51
	120	1.62	0.72	−0.52	0.26	−4.41

Log change was calculated as change in log_10_ CFU/mL from 0 h (CFU_0_) to time t (CFU_t_), where log change = log_10_ (CFU_t_) − log_10_ (CFU_0_). Blue shading indicates enhanced activity and green shading indicates synergy. Enhanced activity was defined as a 1 to <2 log_10_ superior killing for the combination compared to its most active component at the specified time and ≥2 log_10_ below the initial inoculum. Synergy was defined as ≥2 log_10_ bacterial killing for the combination relative to its most active component at the specified time and ≥2 log_10_ below the initial inoculum.

**Table 3:** Clinically representative exposures and pharmacokinetic/pharmacodynamic indices for piperacillin and tobramycin against Pa1281 and CR380 following different dosage regimens.

Isolate, Antibiotic	Regimen	*f*C_max_/*f*C_min_ or *f*C_ss_ (mg/L)	*f*AUC_24_ (mg∗h/L)	*f*C_max_/MIC	*f*T_>MIC_ (%)	*f*T_>4× MIC_ (%)	*f*T_>10× MIC_ (%)	*f*AUC_24_/MIC
Pa1281								
Piperacillin-tazobactam	4 g q4 h ^a^	117/23.1	1477	29.25	100	100	75	369.25
	24 g/day CI ^a^	58	1477		100	100	100	369.25
Tobramycin	7 mg/kg q24 h	24.7/0.0619	112	59.4	70			224
CR380								
Piperacillin-tazobactam	4 g q4 h ^a^	117/23.1	1477	3.66	90	0	0	46.16
	24 g/day CI ^a^	58	1477		100	0	0	46.16
Tobramycin	7 mg/kg q24 h	24.7/0.0619	112	24.7	58			112

^a^ piperacillin dose. All values presented relate to the pharmacokinetics at steady-state for critically ill patients with normal renal clearance. CI, continuous infusion; *f*C_max_ unbound maximal concentration; *f*C_min_ unbound minimal concentration; *f*C_ss_ unbound concentration at steady state; *f*AUC_24_, area under the unbound concentration–time curve over 24 h; *f*C_max_/MIC ratio o*f f*C_max_ to MIC, *f*T_>MIC_, percentage of time that unbound concentration exceeded MIC, *f*T_>4× MIC_ and *f*T_>10× MIC_ percentage o*f* time that unbound concentration exceeded 4× and 10× MIC, respectively; *f*AUC_24_/MIC, ratio o*f f*AUC_24_ to MIC.

## Data Availability

All data are presented in figures and tables as part of the manuscript and Supplementary Materials.

## Supplementary Materials

The following supporting information can be downloaded at: https://www.mdpi.com/article/10.3390/antibiotics11010101/s1, Figure S1: Observed (average ± SE) versus targeted piperacillin and tobramycin concentrations in the dynamic in vitro infection model studies; Table S1: Log change for each treatment as a function of time from static-concentration time-kill studies. Treatments were either piperacillin (Pip)-tazobactam, tobramycin (Tob), or a combination, at the concentrations indicated. Blue shading indicates enhanced activity and green shading indicates synergy.
