# Supplementary material for: Effect of Different Piperacillin-Tazobactam Dosage Regimens on Synergy of the Combination with Tobramycin against Pseudomonas aeruginosa for the Pharmacokinetics of Critically Ill Patients in a Dynamic Infection Model
Source: Antibiotics (Basel). 2022 Jan 13;11(1):101. doi: 10.3390/antibiotics11010101 (PMC8772788; doi:10.3390/antibiotics11010101)
Supplement: Supplementary file 1 [file antibiotics-11-00101-s001.zip › antibiotics-1514224-supplementary.pdf]

antibiotics

MDPI

Article

Effect of Different Piperacillin-Tazobactam Dosage Regimens on Synergy of the Combination with Tobramycin against *Pseudomonas aeruginosa* for the Pharmacokinetics of Critically Ill Patients in a Dynamic Infection Model

Jessica R. Tait [1], Hajira Bilal [2], Kate E. Rogers [1], Yinzhi Lang [3], Tae Hwan Kim [4], Jieqiang Zhou [3], Steven C. Wallis [5], Jürgen B. Bulitta [3], Carl M. J. Kirkpatrick [2], David L. Paterson [5], Jeffrey Lipman [5,6,7,8], Phillip J. Bergen [2], Jason A. Roberts [5,6,7], Roger L. Nation [1] and Cornelia B. Landersdorfer [1,*]

[1] Drug Delivery, Disposition and Dynamics, Monash Institute of Pharmaceutical Sciences, Monash University, Parkville, VIC 3052, Australia; Jessica.Tait@monash.edu (J.R.T.); Kate.Rogers@monash.edu (K.E.R.); Roger.Nation@monash.edu (R.L.N.)
[2] Centre for Medicine Use and Safety, Monash Institute of Pharmaceutical Sciences, Monash University, Parkville, VIC 3052, Australia; hajira.bilal1@monash.edu (H.B.); carl.kirkpatrick@monash.edu (C.M.J.K.); phillip.bergen@monash.edu (P.J.B.)
[3] Department of Pharmacotherapy and Translational Research, College of Pharmacy, University of Florida, Orlando, FL 32827, USA; y.lang@cop.ufl.edu (Y.L.); zhou.jieqiang@cop.ufl.edu (J.Z.); JBulitta@cop.ufl.edu (J.B.B.)
[4] College of Pharmacy, Daegu Catholic University, Gyeongsan 38430, Korea; taehwan.sch@gmail.com
[5] The University of Queensland Center for Clinical Research, The University of Queensland, Brisbane, QLD 4029, Australia; s.wallis@uq.edu.au (S.C.W.); david.antibiotics@gmail.com (D.L.P.); j.lipman@uq.edu.au (J.L.); j.roberts2@uq.edu.au (J.A.R.)
[6] Intensive Care Unit, Royal Brisbane and Women's Hospital, Brisbane, QLD 4029, Australia
[7] Division of Anaesthesiology Critical Care Emergency and Pain Medicine, Nîmes University Hospital, University of Montpellier, 30900 Nîmes, France
[8] Jamieson Trauma Institute, Royal Brisbane and Women's Hospital, Brisbane, QLD 4029, Australia
* Correspondence: Cornelia.Landersdorfer@monash.edu; Tel.: +61-3-990-39011

Supplementary materials

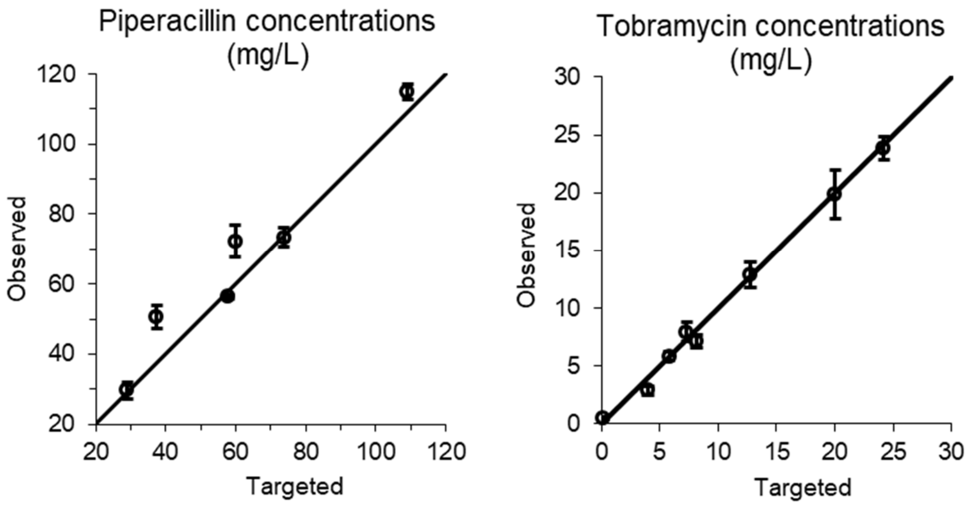

Figure S1. Observed (average[a]±SE) *versus* targeted piperacillin and tobramycin concentrations in the dynamic in vitro infection model studies.

[a] **For piperacillin targeted concentrations of 29.1**, 37.5, 58, 59.9, 73.8 and 109 mg/L, the number of datapoints measured were 8, 17, 40, 6, 16, and 40, respectively. For tobramycin targeted concentrations of 0.159, 4.00, 5.81, 7.32, 8.15, 12.8, 20.0, and 24.2 mg/L, the number of datapoints measured were 3, 3, 5, 5, 7, 8, 7, and 32, respectively.

Table S1. Log change for each treatment as a function of time from static-concentration time-kill studies. Treatments were either piperacillin (Pip)-tazobactam, tobramycin (Tob), or a combination, at the concentrations indicated. Blue shading indicates enhanced activity[a] and green shading indicates synergy [b].

			Log change [$\log_{10}$ (CFU_t) - $\log_{10}$ (CFU_0)]														
**Isolate**	**Initial inoculum ($\log_{10}$CFU/mL)**	**Time (h)**	**Pip 12 mg/L**	**Pip 24 mg/L**	**Pip 75 mg/L**	**Tob 1 mg/L**	**Tob 2 mg/L**	**Tob 8 mg/L**	**Pip 12 + Tob 1 mg/L**	**Pip 24 + Tob 1 mg/L**	**Pip 75 + Tob 1 mg/L**	**Pip 12 + Tob 2 mg/L**	**Pip 24 + Tob 2 mg/L**	**Pip 75 + Tob 2 mg/L**	**Pip 12 + Tob 8 mg/L**	**Pip 24 + Tob 8 mg/L**	**Pip 75 + Tob 8 mg/L**
CR382	5.99	**1.5**	-0.91	-0.82	-0.81	-2.24	-2.76	-4.09	-2.41	-2.56	-2.30	-2.71	-2.24	-2.85	-4.21	-3.44	-3.79
		**3**	-0.52	-1.21	-1.09	-3.00	-3.76	-4.69	-3.04	-331	-2.82	-3.76	-3.61	-3.37	-4.99	-4.99	-5.99
		**6**	-0.20	-0.94	-1.24	-2.82	-3.79	-5.99	-3.12	-3.49	-3.13	-4.29	-3.99	-4.04	-5.99	-5.99	-5.99
		**24**	2.10	1.39	-1.67	2.10	-3.12	-4.39	-2.46	-2.82	-2.63	-3.37	-2.47	-2.92	-4.21	-3.99	-3.85
		**29**	2.19	1.48	-1.56	2.68	-2.04	-4.69	-3.79	-3.23	-3.59	-4.99	-4.15	-4.69	-4.99	-5.99	-4.99
		**48**	2.09	1.47	-1.36	3.39	3.31	-4.99	-2.67	-1.98	-3.18	-4.69	-2.52	-4.39	-5.99	-5.99	-5.99
		**72**	1.70	1.24	-1.24	3.03	3.55	-5.99	-2.68	-2.58	-3.35	-5.99	-5.99	-5.99	-5.99	-4.99	-5.99
CR382	7.38	**1.5**	-0.05	-0.07	-0.05	-0.25	-0.49	-2.60	-0.61	-0.60	-0.71	-1.01	-0.98	-1.10	-2.80	-2.71	-2.71
		**3**	0.54	0.38	0.34	0.38	-0.63	-3.79	-0.73	-1.47	-1.60	-1.76	-2.20	-2.56	-4.26	-3.87	-3.81
		**6**	0.69	0.49	0.29	0.80	-0.40	-4.25	-1.80	-2.59	-2.76	-2.47	-2.98	-3.06	-4.70	-4.28	-4.19
		**24**	1.31	1.47	1.43	1.50	1.56	-1.72	1.29	0.71	-2.35	0.88	-0.36	-2.59	-3.78	-3.40	-3.17
		**29**	1.28	1.68	1.51	1.49	1.05	-0.65	0.88	0.63	-1.86	0.70	-0.28	-3.34	-5.26	-5.38	-5.20
		**48**	0.84	0.83	0.80	1.60	1.14	1.43	0.82	1.11	-1.58	0.35	-0.59	-1.52	-4.06	-3.82	-3.63
		**72**	1.18	0.79	0.89	0.43	1.32	0.69	0.05	0.96	-1.36	1.02	-0.44	-1.43	-4.95	-4.29	-4.93
CR379	5.87	**1.5**	-0.49	-0.38	-0.55	-2.10	-2.06	-3.32	-2.22	-2.08	-2.36	-2.90	-2.28	-3.19	-3.39	-3.97	-3.72
		**3**	0.69	0.20	-0.38	-3.05	-4.17	-4.57	-3.51	-3.45	-4.09	-4.87	-4.87	-4.39	-5.87	-4.87	-5.87
		**6**	1.07	-0.23	-2.19	-3.75	-4.57	-5.87	-3.97	-4.17	-4.39	-5.87	-5.87	-5.87	-5.87	-5.87	-5.87

		**24**	1.17	1.14	-2.02	2.34	-4.27	-5.87	-2.94	-3.27	-4.27	-4.57	-5.87	-4.17	-5.87	-5.87	-5.87
		**29**	1.66	1.12	-1.06	3.05	-3.79	-5.87	-3.39	-3.25	-3.69	-3.91	-5.87	-3.57	-5.87	-5.87	-5.87
		**48**	1.42	0.72	-0.74	3.14	3.01	-4.87	-1.19	-2.29	-3.64	-5.87	-5.87	-4.87	-5.87	-5.87	-5.87
		**72**	1.80	1.18	-0.28	3.22	3.24	-5.87	-0.19	-1.08	-3.42	-4.87	-5.87	-5.87	-5.87	-5.87	-5.87
CR379	7.57	**1.5**	-0.14	-0.27	-0.23	-0.52	-1.45	-3.60	-0.82	-0.70	-0.88	-1.79	-1.71	-1.77	-3.37	-3.48	-3.46
		**3**	0.39	0.16	0.07	-0.62	-3.01	-4.91	-1.49	-1.90	-2.27	-3.91	-3.45	-3.70	-4.51	-4.90	-5.14
		**6**	0.61	0.38	0.15	0.17	-2.65	-4.82	-1.87	-3.00	-3.17	-3.50	-3.38	-3.57	-4.96	-4.89	-5.04
		**24**	0.19	-0.34	-0.82	1.26	1.05	-2.90	-0.01	-1.82	-2.45	-2.51	-2.60	-2.48	-3.23	-2.93	-3.05
		**29**	0.19	-0.79	-1.57	1.15	1.30	-5.14	0.16	-1.65	-2.66	-2.69	-2.75	-2.72	-5.67	-5.57	-5.97
		**48**	-0.15	-0.23	-0.95	1.18	1.34	-2.19	-0.37	-0.08	-2.44	-1.17	-1.69	-2.39	-4.30	-4.79	-5.05
		**72**	0.05	-0.63	-1.43	0.73	0.85	1.19	0.22	-0.05	-2.62	0.77	-1.63	-2.88	-7.57	-5.97	-5.39

		**Time (h)**	**Pip 12 mg/L**	**Pip 24 mg/L**	**Pip 75 mg/L**	**Tob 1mg/L**	**Tob 2 mg/L**	**Tob 8 mg/L**	**Pip 12 + Tob 1 mg/L**	**Pip 24 + Tob 1 mg/L**	**Pip 75 + Tob 1 mg/L**	**Pip 12 + Tob 2 mg/L**	**Pip 24 + Tob 2 mg/L**	**Pip 75 + Tob 2 mg/L**	**Pip 12 + Tob 8 mg/L**	**Pip 24 + Tob 8 mg/L**	**Pip 75 + Tob 8 mg/L**
CR380	5.80	**1.5**	-0.34	-0.46	-1.09	-1.70	-2.53	-4.20	-2.03	-2.08	-2.22	-2.74	-3.09	-3.07	-3.76	-3.90	-4.02
		**3**	0.26	0.01	-1.10	-2.53	-3.63	-5.80	-3.34	-2.96	-3.35	-3.35	-4.20	-4.32	-4.80	-4.80	-4.80
		**6**	1.93	0.83	-1.35	-3.23	-3.96	-5.80	-3.17	-3.18	-3.50	-3.85	-5.80	-5.80	-4.80	-5.80	-5.80
		**24**	1.80	1.31	-0.81	2.81	-1.03	-5.80	-0.84	-2.50	-2.85	-2.66	-3.48	-3.76	-4.80	-5.80	-5.80
		**29**	1.81	1.06	-0.78	3.31	0.13	-5.80	-0.56	-3.96	-4.50	-4.50	-5.80	-5.80	-5.80	-5.80	-5.80
		**48**	1.60	1.18	-0.22	3.60	3.28	-5.80	2.29	-2.81	-3.11	-3.26	-4.20	-5.80	-5.80	-5.80	-5.80
		**72**	1.56	1.44	0.14	2.60	2.61	-5.80	2.18	-2.96	-3.04	-3.39	-3.96	-5.80	-5.80	-5.80	-5.80
CR380	7.28	**1.5**	0.10	-0.15	-0.27	-1.05	-1.69	-3.04	-1.39	-1.34	-1.57	-2.06	-2.04	-2.08	-2.98	-3.15	-2.98
		**3**	0.25	-0.07	-0.17	-0.76	-1.96	-4.15	-1.41	-1.34	-2.06	-2.54	-2.99	-3.13	-3.54	-3.96	-3.87
		**6**	0.58	0.31	-0.65	0.49	-1.96	-4.44	-1.10	-1.50	-2.59	-2.76	-3.26	-3.58	-4.08	-4.19	-4.10

		**24**	0.11	-0.84	-1.81	1.02	1.73	-3.44	0.47	-0.96	-2.26	-0.56	-2.84	-3.07	-3.29	-3.57	-3.68
		**29**	0.54	0.11	-1.39	1.79	1.77	-2.63	0.18	-0.92	-3.28	-0.18	-4.00	-4.27	-5.58	-5.38	-5.68
		**48**	-0.25	-0.82	-1.15	1.72	1.65	1.78	0.16	-0.54	-3.04	0.72	-3.09	-3.50	-5.58	-4.90	-4.98
		**72**	-0.21	-0.67	-1.28	1.51	1.89	1.84	0.26	0.32	-2.25	0.50	-1.58	-2.96	-4.59	-5.28	-5.08
Pa1281	6.16	**1.5**	-0.11	-0.11	-0.20	-3.12	-3.75	-4.86	-3.41	-3.21	-3.80	-3.91	-4.02	-3.78	-5.16	-6.16	-5.16
		**3**	-1.66	-1.58	-1.76	-3.69	-4.86	-6.16	-3.96	-4.56	-4.56	-5.16	-5.16	-5.16	-6.16	-6.16	-6.16
		**6**	-2.69	-2.78	-3.56	-4.86	-6.16	-6.16	-6.16	-6.16	-6.16	-6.16	-6.16	-6.16	-6.16	-6.16	-6.16
		**24**	1.16	-0.64	-3.15	-2.02	-6.16	-6.16	-6.16	-5.16	-6.16	-6.16	-6.16	-6.16	-6.16	-6.16	-6.16
		**29**	2.05	1.25	-3.55	-0.61	-6.16	-6.16	-6.16	-6.16	-6.16	-6.16	-6.16	-6.16	-6.16	-6.16	-6.16
		**48**	2.55	2.05	-2.30	2.85	-0.54	-6.16	-6.16	-6.16	-6.16	-6.16	-6.16	-6.16	-6.16	-6.16	-6.16
		**72**	2.81	2.65	0.25	3.06	2.20	-6.16	-6.16	-6.16	-6.16	-6.16	-6.16	-6.16	-6.16	-6.16	-6.16
Pa1281	7.46	**1.5**	0.26	0.09	-0.14	-1.62	-3.47	-4.71	-2.80	-1.66	-2.91	-3.44	-3.67	-3.88	-5.76	-6.46	-7.46
		**3**	0.27	0.26	0.22	-3.19	-3.89	-5.68	-4.08	-3.86	-3.93	-4.48	-4.71	-5.21	-7.46	-7.46	-7.46
		**6**	0.11	0.06	-0.12	-3.74	-4.89	-6.46	-4.53	-4.45	-4.64	-5.32	-5.56	-6.16	-7.46	-7.46	-7.46
		**24**	-0.85	-0.67	-0.75	0.99	-1.11	-7.46	-1.86	-3.62	-3.62	-5.35	-5.38	-5.16	-7.46	-7.46	-7.46
		**29**	-0.52	-0.46	-0.83	1.45	0.36	-7.46	-3.85	-7.46	-7.46	-7.46	-7.46	-7.46	-7.46	-7.46	-7.46
		**48**	-0.14	-0.19	-0.49	1.60	1.36	-7.46	-0.23	-7.46	-7.46	-7.46	-7.46	-7.46	-7.46	-7.46	-7.46
		**72**	0.42	0.41	-0.37	1.71	1.60	-4.15	1.03	-7.46	-7.46	-7.46	-7.46	-7.46	-7.46	-7.46	-7.46

Log change was calculated as change in $\log_{10}$ CFU/mL from 0 h (CFU_0) to time t (CFU_t), where log change = $\log_{10}$ (CFU_t) - $\log_{10}$ (CFU_0). [a]enhanced activity was defined as a 1 to <2 $\log_{10}$ superior killing for the combination compared to its most active component at the specified time and ≥2 $\log_{10}$ below the initial inoculum; [b]synergy was defined as ≥2 $\log_{10}$ bacterial killing for the combination relative to its most active component at the specified time and ≥2 $\log_{10}$ below the initial inoculum.
